# Melatonin promotes triacylglycerol accumulation via MT2 receptor during differentiation in bovine intramuscular preadipocytes

**DOI:** 10.1038/s41598-017-12780-y

**Published:** 2017-11-08

**Authors:** Wucai Yang, Keqiong Tang, Yaning Wang, Yingying Zhang, Linsen Zan

**Affiliations:** 1 College of Animal Science and Technology, Northwest A&F University, Yangling, Shaanxi 712100 China; 2 College of Veterinary Medicine, Northwest A&F University, Yangling, Shaanxi 712100 China

## Abstract

Melatonin (N-acetyl-5-methoxytryptamine) is a derivative of tryptophan which is produced and secreted mainly by the pineal gland and regulates a variety of important central and peripheral actions. To examine the potential effects of melatonin on the proliferation and differentiation of bovine intramuscular preadipocytes (BIPs), BIPs were incubated with different concentrations of melatonin. Melatonin supplementation at 1 mM significantly increased peroxisome proliferator-activated receptor γ (PPARγ), CCAAT/enhancer-binding protein (C/EBP) β, and C/EBPα expression and promoted the differentiation of BIPs into adipocytes with large lipid droplets and high cellular triacylglycerol (TAG) levels. Melatonin also significantly enhanced lipolysis and up-regulated the expression of lipolytic genes and proteins, including hormone sensitive lipase (HSL), adipocyte triglyceride lipase (ATGL), and perilipin 1 (PLIN1). Moreover, melatonin reduced intracellular reactive oxygen species (ROS) levels by increasing the expression levels and activities of superoxide dismutase 1 (SOD1) and glutathione peroxidase 4 (GPX4). Finally, the positive effects of melatonin on adipogenesis, lipolysis, and redox status were reversed by treatment with luzindole, anantagonist of nonspecific melatonin receptors 1 (MT1) and 2 (MT2), and 4-phenyl-2-propionamidotetraline (4P-PDOT), a selective MT2 antagonist. These results reveal that melatonin promotes TAG accumulation via MT2 receptor during differentiation in BIPs.

## Introduction

Intramuscular fat (IMF) content, which is termed marbling when visually assessed, plays a critical role in the experience of consuming beef, and a positive relationship between IMF and palatability (meat color, flavor, juiciness, and tenderness) is well established^1,2^. High-marbling cuts can command a price premium in many countries and grading systems, such as in China^3^, Japan^4^, and the United States^5^. Previous studies have demonstrated that variability in IMF content is determined by the number and size of intramuscular adipocytes^6,7^. Thus, given the cost of intensively feeding cattle to improve IMF levels, it is of relevance to understand the mechanism of bovine intramuscular preadipocytes (BIPs) proliferation and differentiation.

Melatonin (N-acetyl-5-methoxytryptamine), a derivative of tryptophan, is produced and secreted at night mainly by the pineal gland in mammals. As a multifunctional molecule, melatonin regulates a variety of important central and peripheral actions related to circadian rhythms, visual, reproduction and neuroendocrine in mammals^8–10^. Given the central role of melatonin in reproductive physiology, exogenous melatonin has been applied to control reproductive activity in farm animals^11,12^. Additionally, recent studies have revealed important regulatory roles of melatonin in body fat mass and energy metabolism regulation^13–15^. Pinealectomized rats exhibit increased accumulation of adipose depots as a result of reduced levels of circulating melatonin^16^. Exogenous melatonin inhibited both body weight gain and abdominal fat deposition in laboratory animals^7–19^, but promoted fat deposition in the rib and longissimus muscle in post-pubertal heifers^20^. However, the actual contribution of melatonin to adipose tissue growth is currently unknown.

At present, the potential role of melatonin in adipogenesis has been extensively studied in the 3T3-L1 cell line, but contradictory results have been reported. Some studies demonstrated that melatonin suppresses adipogenesis by down-regulating PPARγ, C/EBPβ, and C/EBPα in 3T3-L1 cells^13^. In sharp contrast, other studies show that melatonin stimulates adipocyte differentiation in 3T3-L1 cells and increases intracytoplasmic ATG accumulation in murine fibroblasts by up-regulating PPARγ, C/EBPα, and C/EBPβ^21,22^. On the other hand, some studies have found that melatonin stimulates the differentiation of 3T3-L1 into adipocytes but also promotes lipolysis and results in small lipid droplets^23^. To summarize, the contribution of melatonin to the regulation of adipogenesis remains uncertain. Moreover, recent advances indicate that the regulatory mechanism underlying adipogenesis might differ among animal species^24–27^; therefore, research on the 3T3-L1cell line cannot truly reflect the process of adipogenesis in bovines.

In mammals, numerous physiological roles of melatonin are mediated via activation of two high-affinity G protein-coupled receptors, MT1 and MT2^28,29^, which are expressed both singly and together in various tissues with different expression profiles^30–32^. Regarding the mediation of melatonin functions, MT1 and MT2 receptors appear to vary among different tissues and cell types, and even within the same cell type^29,33^. Morgan *et al*.^34^ found that MT1 receptor activation inhibits cAMP accumulation in hypophyseal pars tuberalis^34^ but stimulates cAMP production in COS-7 and SH-SY5Y cells^35,36^. The inhibitory effects of melatonin on cAMP accumulation were likely mediated only through MT2 receptors in human PAZ6 adipocytes and HEK293 cells^26,37^. Furthermore, MT1 and MT2 receptors are co-expressed in the rat suprachiasmatic nucleus (SCN) but regulate distinct aspects of SCN physiology^38^. At present, studies related to the melatonin receptor-mediated effects on preadipocyte differentiation in mammals remain elusive.

Considering the data summarized above, we hypothesized that melatonin would induce the adipogenesis on BIPs and that the effects would be mediated by the membrane receptors. To test this hypothesis, luzindole, a nonspecific MT1/MT2 antagonist, and 4P-PDOT, a selective MT2 antagonist, were used to identify which receptor is involved in these actions. We demonstrate for the first time that melatonin promotes BIPs differentiation into adipocytes with larger lipid droplets by increasing the expression of adipogenic molecules via the MT2 receptor. In addition, melatonin enhances lipolysis and reduces intracellular ROS levels by up-regulating the expression of lipolytic and antioxidant molecules via the MT2 receptor.

## Results

### Melatonin promotes TAG accumulation during differentiation in BIPs

**Figure 1:**
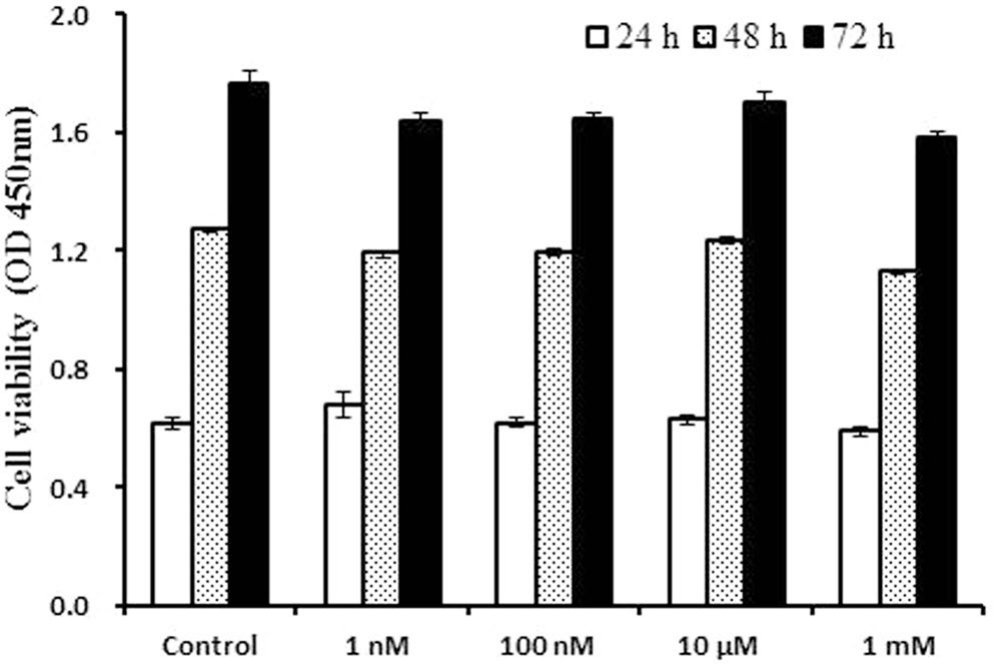
Effects of different concentrations of melatonin on the proliferation of BIPs. Cell viability was determined by CCK-8 assay at the three time points. Data shown are the mean ± S.E.M. of the ratio for light absorbance at 450 nm. BIPs = bovine intramuscular preadipocytes.

**Figure 2:**
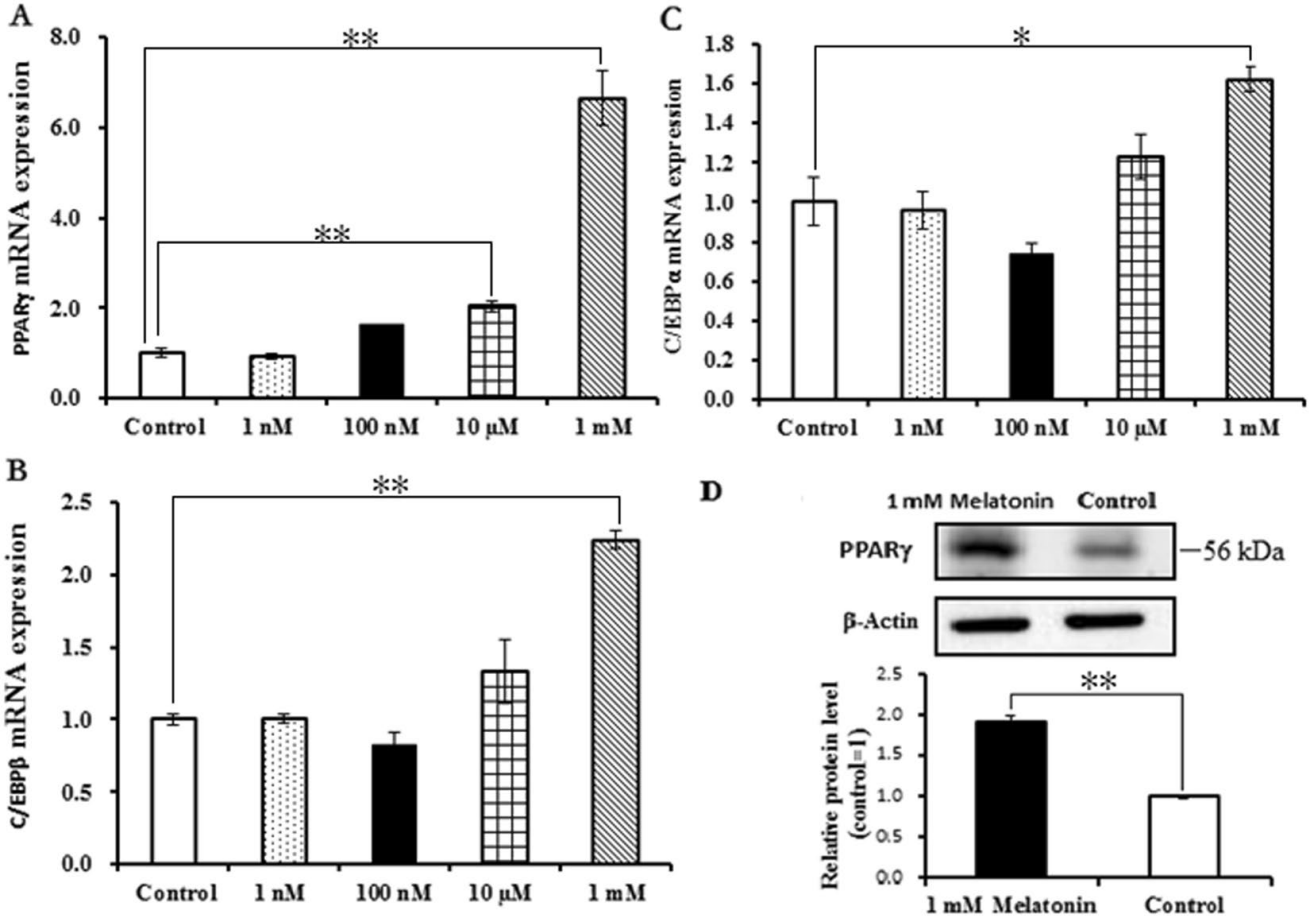
Effects of different concentrations of melatonin on the differentiation of BIPs. (**A**) The mRNA expression of adipogenic master regulator PPARγ; (**B**) the mRNA expression of adipogenic master regulator C/EBPβ; (**C**) the mRNA expression of adipogenic master regulator C/EBPα; (**D**) Protein level of PPARγ was quantitated using densitometry and normalized to β-actin levels. The expression of adipogenic master regulator genes mRNA were normalized to housekeeping genes β-actin and GAPDH. Values are presented as the means ± S.E.M. “**”represents significant differences *P* < 0.01. PPARγ = peroxisome proliferator-activated receptorγ; C/EBPβ = CCAAT/enhancer-binding protein β; C/EBPα = CCAAT/enhancer-binding protein α.

**Figure 3:**
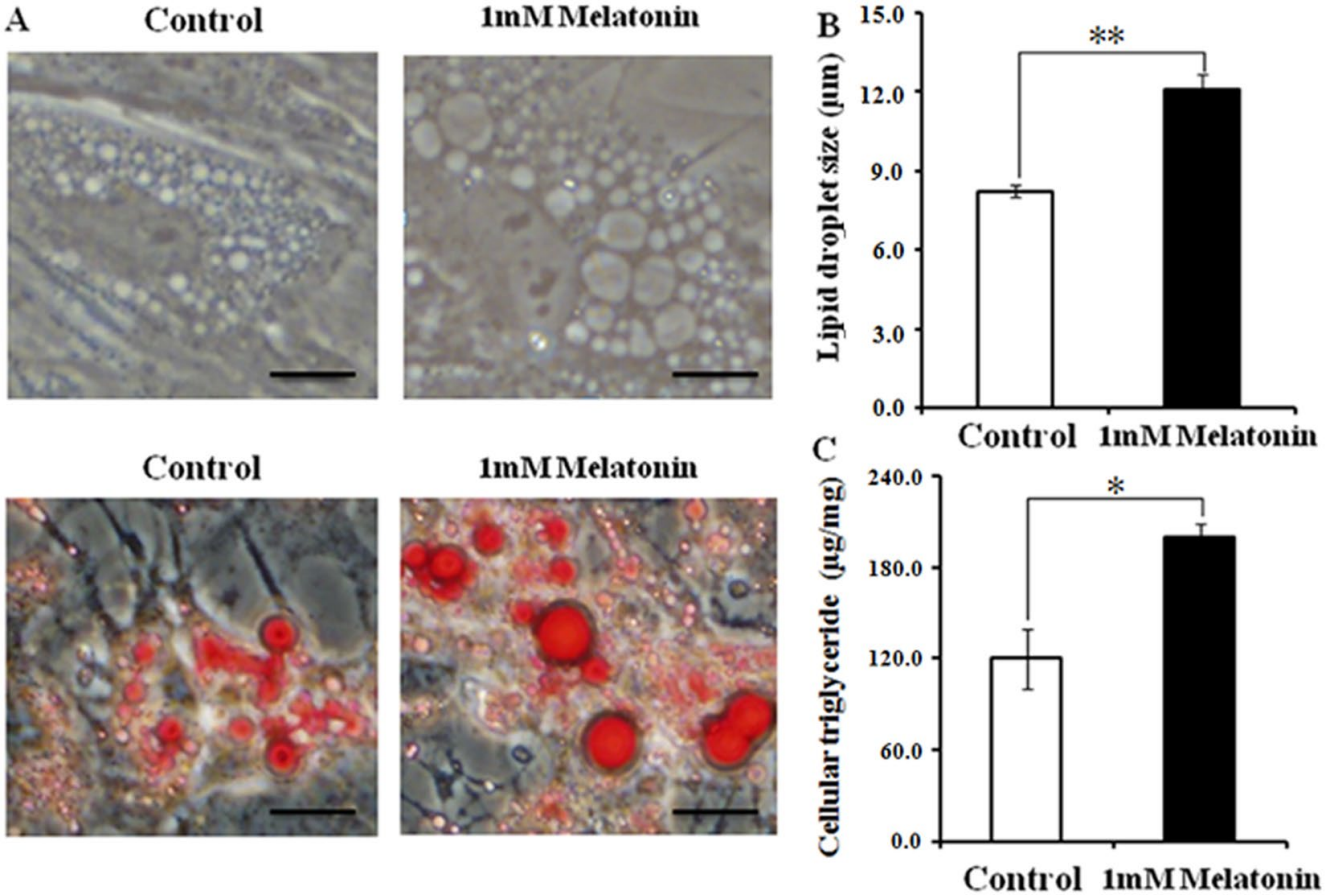
Effect of 1 mM melatonin on morphological changes and lipid accumulation in BIPs. (**A**) Representative images of unstained or oil-red O stained cells after 8 days of differentiation; (**B**) the average size of 100 lipid droplets per sample was measured on Image J software; (**C**) effects of melatonin on lipid accumulation. Scale bar = 50 μm. Values are presented as the means ± S.E.M. “*”represents significant differences, *P* < 0.05; “**”represents significant differences *P* < 0.01. BIPs = bovine intramuscular preadipocytes.

Melatonin did not significantly affect the proliferation of BIPs (Fig. 1). To assess whether melatonin positively affects BIP differentiation, we treated cells with different melatonin concentrations (1 nM, 100 nM, 10 µM, and 1 mM). As shown in Fig. 2A,D, melatonin at 1 mM significantly increased the expression of PPARγ, a master regulator of adipogenesis, at the mRNA and protein levels. In addition, the expression levels of C/EBPβ and C/EBPα were significantly up-regulated in the 1 mM melatonin-treated cells (Fig. 2B,C). We also measured the lipid droplet size and cellular TAG content in adipocytes; melatonin at 1 mM significantly increased the lipid droplet size (Fig. 3A,B) and resulted in a higher TAG level (Fig. 3C) compared with the control cells.

### Melatonin promotes adipocyte lipolysis and reduces intracellular ROS levels

**Figure 4:**
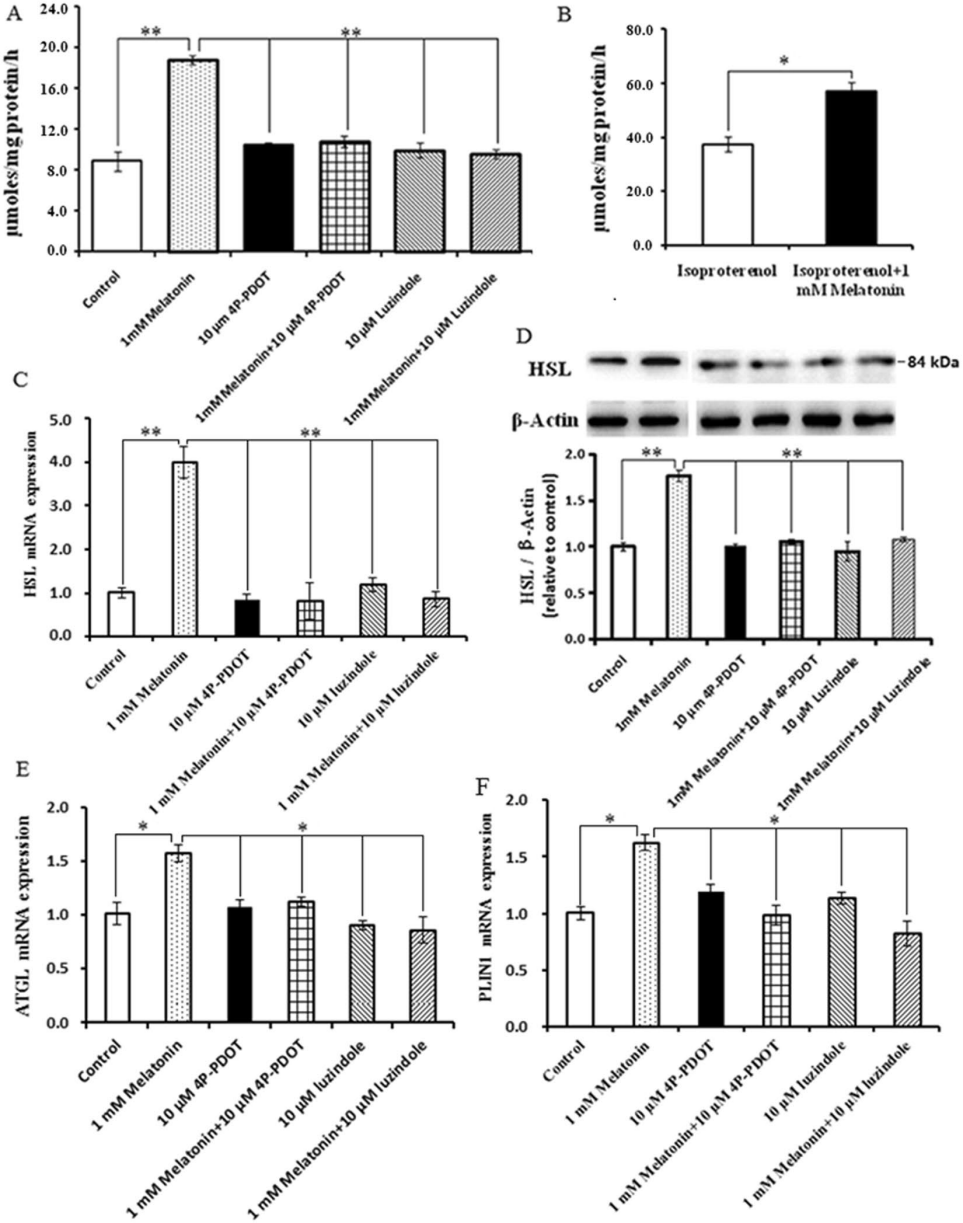
Effect of luzindole or 4P-PDOT on melatonin-induced lipolytic changes in BIPs. (**A**) Effect of luzindole and 4P-PDOT on melatonin-induced adipocyte lipolysis; (**B**) BIPs differentiated over 8 days were incubated in fresh medium with or without 10 μM isoproterenol; (**C**–**E**) the mRNA expression of lipolytic related genes (ATGL, HSL, and PLIN1); (**F**) protein level of HSL was quantitated using densitometry and normalized to β-actin levels. The medium was collected to detect the glycerol release, a measure of lipolysis, using the Adipolysis Assay Kit. Lipolysis was calculated in nmol per mg total protein per h. The mRNA expressions were normalized to housekeeping genes β-actin and GAPDH. Values are presented as the means ± S.E.M. “*”represents significant differences, *P* < 0.05; “**”represents significant differences *P* < 0.01. 4P-PDOT = 4-phenyl-2-propionamidotetraline; BIPs = bovine intramuscular preadipocytes; HSL = hormone sensitive lipase; ATGL = adipocyte triglyceride lipase; PLIN1 = perilipin 1.

**Figure 5:**
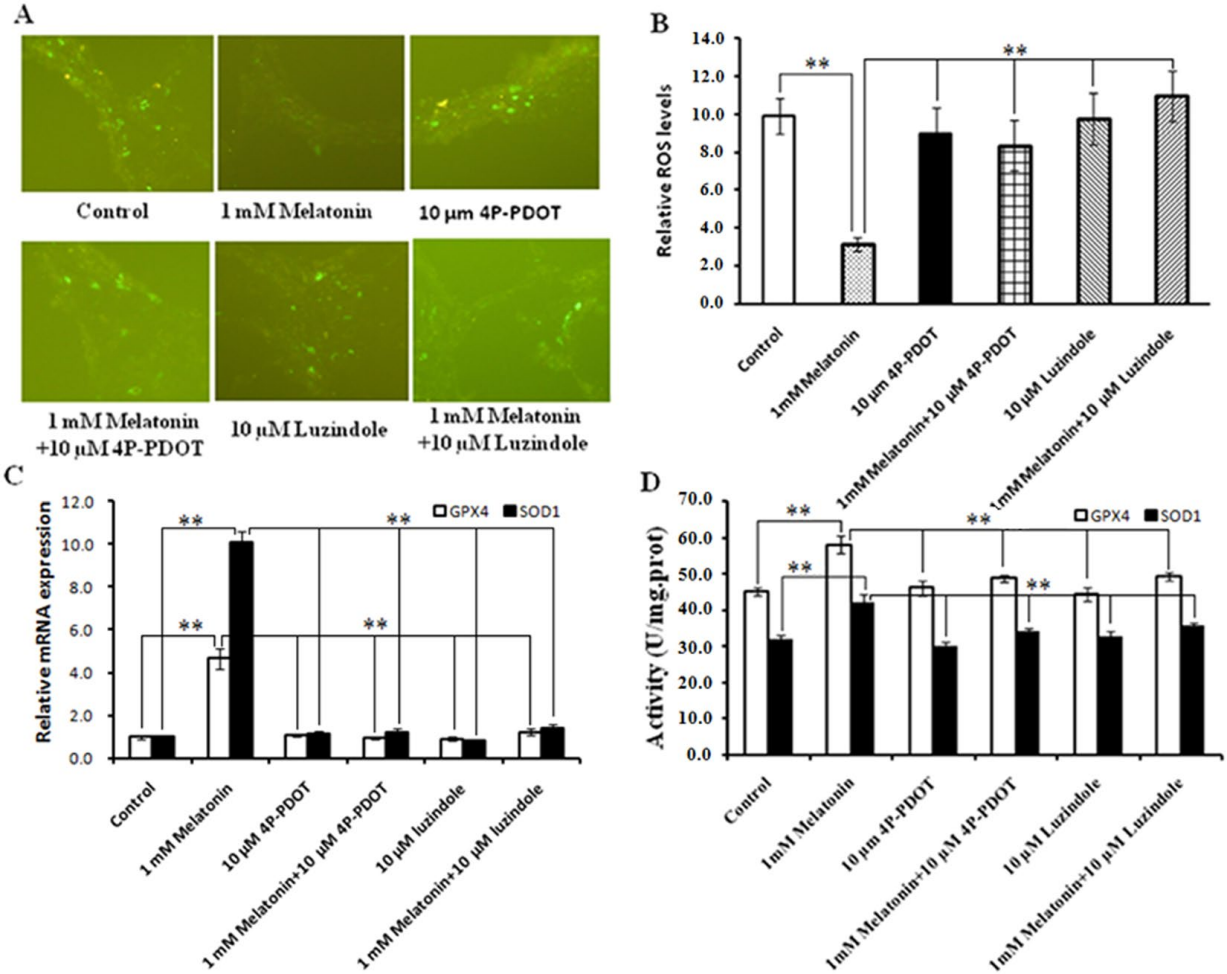
Effect of luzindole or 4P-PDOT on melatonin-induced ROS production in BIPs. (**A**) ROS staining (green fluorescence) in BIPs after differentiation for 8 days with or without melatonin, luzindole or 4P-PDOT; (**B**) effect of luzindole and 4P-PDOT on melatonin-induced ROS production; (**C**) the mRNA expression of antioxidant genes (SOD1 and GPX4); (**D**) the activities of antioxidant enzymes SOD1 and GPX4. The mRNA expression of antioxidant genes were normalized to housekeeping genes β-actin and GAPDH. Values are presented as the means ± S.E.M. “**”represents significant differences *P* < 0.01. BIPs = bovine intramuscular preadipocytes; 4P-PDOT = 4-phenyl-2-propionamidotetraline; SOD1 = superoxide dismutase 1; GPX4 glutathione peroxidase 4.

The glycerol release was measured to determine whether the melatonin-induced increases in the size of lipid droplets and TAG level are associated with reduced lipolysis. As noted in Fig. 4A, basal lipolysis following 1 mM melatonin treatment was increased 2.33-fold compared to the control (P = 0.001) and accompanied by a marked increase in HSL at the mRNA and protein levels (Fig. 4C,D). Moreover, 1 mM melatonin increased ATGL and PLIN1 mRNA expression, which are both related to lipid droplets (Fig. 4E,F). We then quantified the intracellular ROS levels and the expression and activity of the antioxidant SOD1 and GPX4 (Fig. 5). 1 mM melatonin supplements significantly reduced the intracellular ROS levels of intramuscular adipocytes compared with the control (Fig. 5A,B). When cells were treated with 1 mM melatonin, SOD1 and GPX4 mRNA expression were significantly up-regulated and the activities of these antioxidant enzymes significantly increased compared with the controls (Fig. 5C,D).

### The expression profiles of MT1 and MT2 during BIPs differentiation

**Figure 6:**
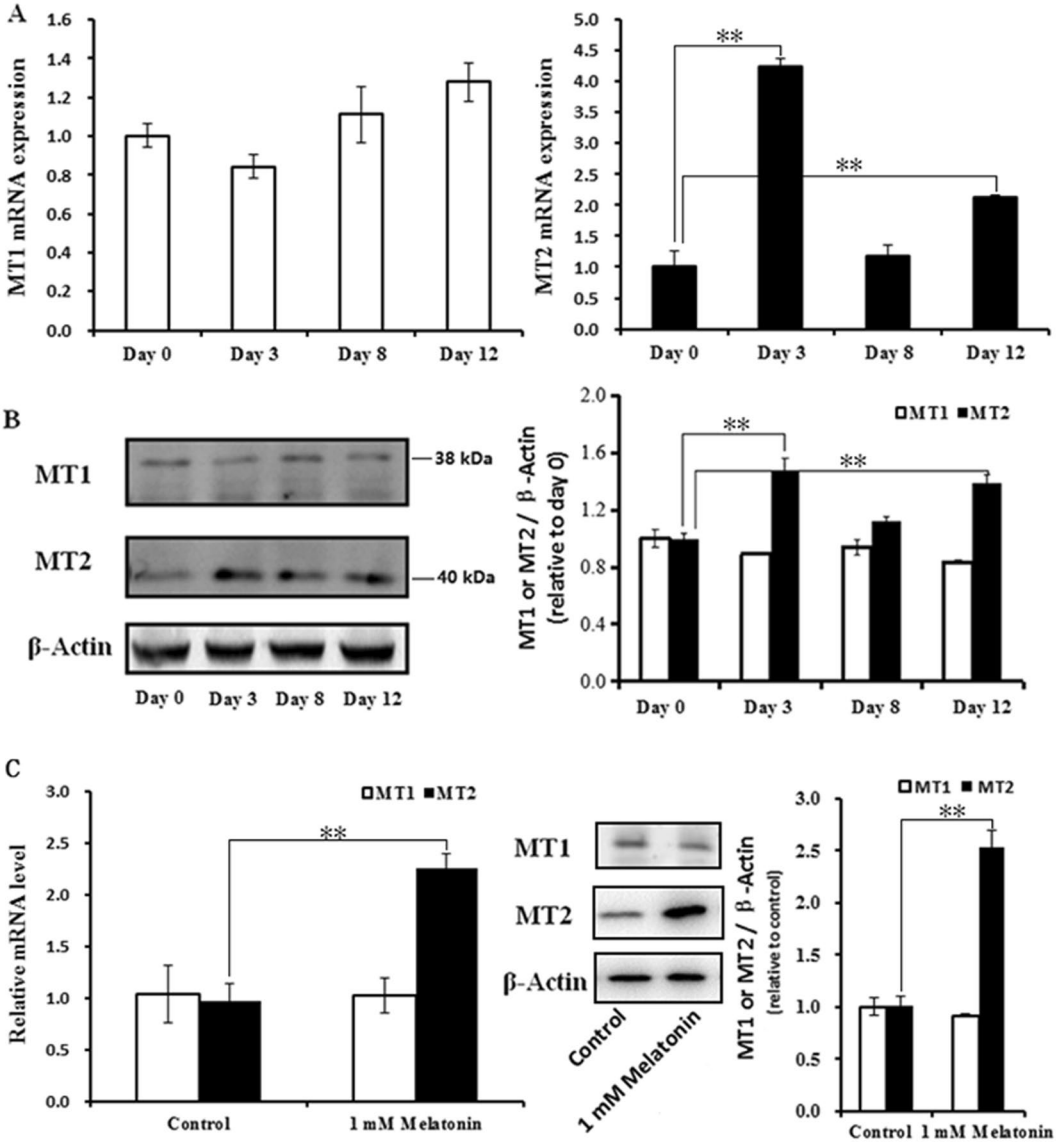
Expression profiles of MT1 and MT2 at different stages of BIP differentiation. (**A**) The mRNA expression of the MT1 and MT2 genes at 3, 8 and 12 d of BIPs differentiation; (**B**) proteins level of MT1 and MT2 were quantitated using densitometry and normalized to β-actin levels; (**C**) Effect of melatonin on the mRNA and protein expression of the MT1 and MT2 genes at 8 d of BIPs differentiation. The expression of MT1 and MT2 mRNA were normalized to housekeeping genes β-actin and GAPDH. Protein level of MT1 and MT2 were quantitated using densitometry and normalized to β-actin levels. Values are presented as the means ± S.E.M. “**”represents significant differences *P* < 0.01. MT1 = melatonin receptors 1; MT2 = melatonin receptors 2; BIPs = bovine intramuscular preadipocytes.

The expression levels of MT1 and MT2 were detected at different stages of BIP differentiation. The results show that the expression of MT2 was significantly up-regulated at 3 and 12 d of BIPs differentiation compared with untreated cells (Fig. 6A), whereas no significant difference in MT1 expression was noted during BIPs differentiation (Fig. 6A). Western blotting analysis with specific antibodies also confirmed the expression profiles of MT1 and MT2 during BIP differentiation (Fig. 6B). Furthermore, 1 mM melatonin significantly increased the expression of MT2 at the mRNA and protein levels during BIPs differentiation (Fig. 6C), whereas no significant difference in MT1 expression was noted (Fig. 6C).

### Role of the MT1 and MT2 on mediating the melatonin effects during BIPs differentiation

**Figure 7:**
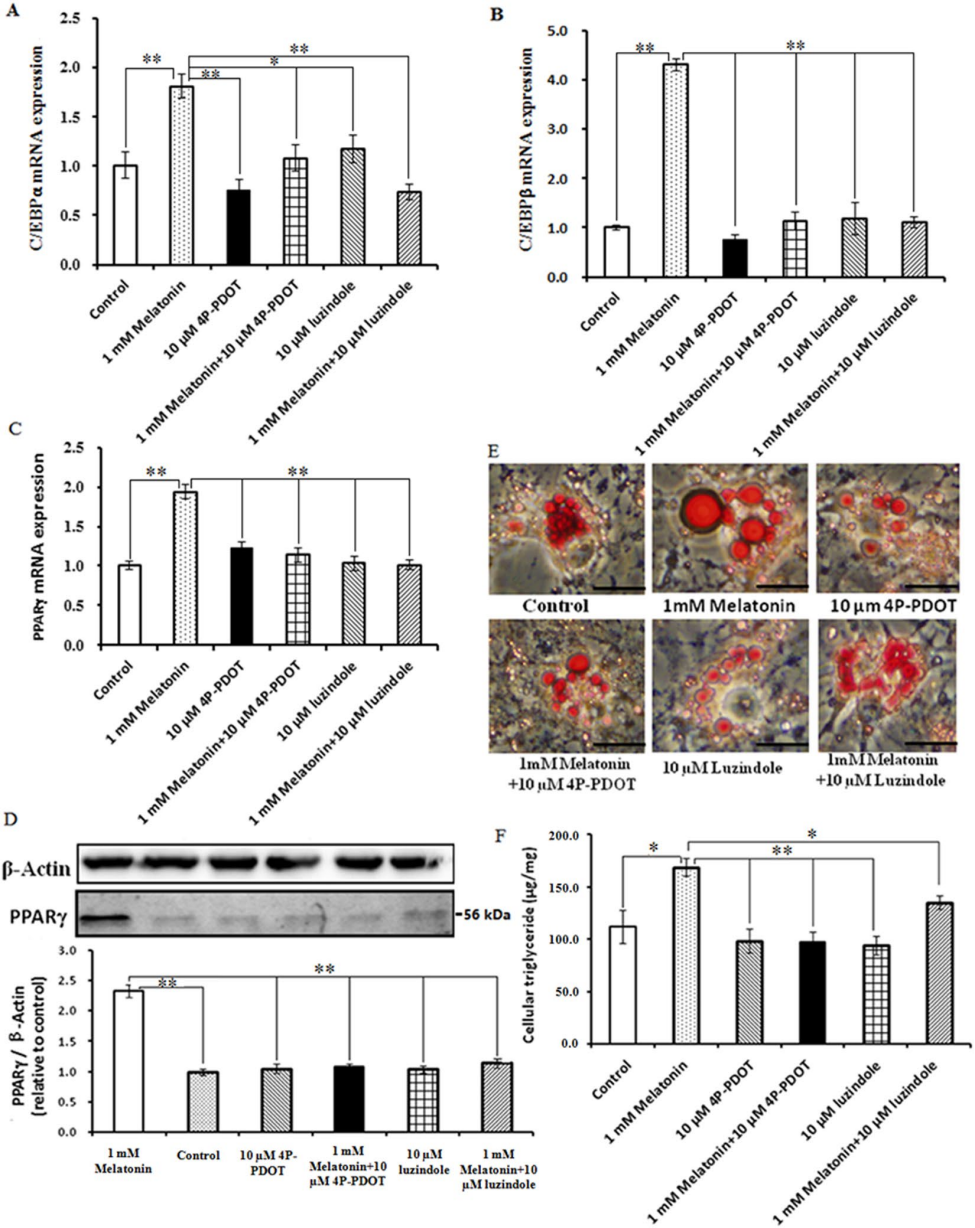
Effect of luzindole or 4P-PDOT on melatonin-induced morphological changes and lipid accumulation in BIPs. (**A**–**C**) Effect of luzindole or 4P-PDOT on melatonin-induced mRNA expression of adipogenic master regulators PPARγ, C/EBPβ, and C/EBPα; (**D**) protein level of PPARγ was quantitated using densitometry and normalized to β-actin levels; (**E**) representative images of oil-red O stained cells after 8 days of differentiation in different treatments; (**F**) effects of luzindole or 4P-PDOT on melatonin-induced lipid accumulation. The expression of adipogenic master regulator genes mRNA were normalized to housekeeping genes β-actin and GAPDH. Scale bar = 50 μm. Values are presented as the means ± S.E.M. “*”represents significant differences, *P* < 0.05; “**”represents significant differences *P* < 0.01. 4P-PDOT = 4-phenyl-2-propionamidotetraline; BIPs = bovine intramuscular preadipocytes; PPARγ = peroxisome proliferator-activated receptor γ; C/EBPβ = CCAAT/enhancer-binding proteinβ; C/EBPα = CCAAT/enhancer-binding proteinα.

Luzindole and 4P-PDOT were used to determine whether melatonin effects are mediated through its specific receptors MT1 and MT2. The results show that the positive effects of 1 mM melatonin on PPARγ, C/EBPβ, and C/EBPα expression are reversed by treatment with 10 µM 4P-PDOT or luzindole (Fig. 7). PPARγ, C/EBPβ and C/EBPα mRNA expression were significantly down-regulated in the 1 mM melatonin plus 10 µM luzindole and 4P-PDOT groups compared with the 1 mM melatonin treated cells (Fig. 7A–C), and western blot analysis revealed that the PPARγ protein levels were consistent with the mRNA expression response (Fig. 7D). Furthermore, the addition of 10 µM luzindole or 4P-PDOT prevented 1 mM melatonin from promoting lipid droplet formation and TAG synthesis. As shown in Fig. 7E,F, 1 mM melatonin plus 10 µM luzindole or 4P-PDOT significantly suppressed cellular TAG accumulation and lipid droplet formation compared with the 1 mM melatonin treated cells. Basal lipolysis was significantly decreased in the 1 mM melatonin plus 10 µM luzindole and 1 mM melatonin plus 4P-PDOT groups compared with the 1 mM melatonin treated cells (Fig. 4A) and was accompanied by a marked down-regulation of HSL, ATGL, and PLIN1 (Fig. 4C–E). Moreover, the addition of 10 µM luzindole or 4P-PDOT prevented the positive effect of 1 mM melatonin on intracellular ROS levels. As noted in Fig. 5A,B, 1 mM melatonin plus luzindole or 4P-PDOT resulted in higher levels of intracellular ROS compared with the 1 mM melatonin treated cells. Consistent with these findings, the increase in ROS levels induced by the addition of luzindole or 4-P-PDOT significantly reduced SOD1 and GPX4 mRNA expression levels and activities (Fig. 5C,D). These results indicate that only MT2 activation is involved in these functions.

## Discussion

Previous studies demonstrated that the role of melatonin in the regulation of cell proliferation dependents on concentration and exposure time as well as cell type and species^39,40^. For example, melatonin has no significant effect on bovine granulosa cell proliferation^41^ but significantly promotes the proliferation of porcine granulosa cells^42^. Zwirska-Korczala *et al*. (2005) reported that melatonin at 1 mM and 10 nM significantly stimulated 3T3-L1 cell proliferation^43^. In contrast, in our study, melatonin at 1 nM, 100 nM, 10 µM and 1 mM had no significant effect on the proliferation of BIPs. This observation may be due to the differences in cell type and species^39–42^.

At present, the role of melatonin in adipogenesis has been extensively studied in hMSCs and 3T3-L1 cell lines, and it has been found that melatonin regulates adipogenesis through sequential activation of the adipogenic master regulators, the PPARγ and C/EBP family members^44,45^. However, contradictory results have also been reported. Some studies have found that melatonin suppresses adipogenesis by down-regulating PPARγ, C/EBPβ, and C/EBPα in 3T3-L1and hMSCs cells^13,46,47^. In contrast, other studies suggest that melatonin works as a positive regulator of adipogenesis by up-regulating the expression of PPARγ and C/EBPα^21,22^. Here, our results confirm that 1 mM melatonin promotes the adipocyte differentiation of BIPs through up-regulating PPARγ, C/EBPβ, and C/EBPα expression. Meantime, we found that the optimal melatonin concentration 1 mM is much higher than the physiological serum concentration of melatonin^8^. This observation is consistent with the finding reported by Pang *et al*.^48^ that melatonin at 1 mM significantly improved the semen quality of bovine^48^. The result indicates that melatonin’s effect at physiological concentrations *in vitro* may be specific only for certain types of cells^49^.

The process of adipocyte lipolysis is critically governed in a manner dependent on the lipases and proteins associated with lipid droplets, including ATGL, HSL, and PLIN1. HSL and ATGL are the major rate-limiting enzymes in adipocyte lipolysis, which coordinately catabolize stored triglycerides^50,51^. Sztalryd *et al*. (2003) confirmed that PLIN1 is required to elicit HSL translocation at the surface of lipid droplets and indicated that lipolysis is a concerted reaction mediated by both protein kinase A-phosphorylated HSL and PLIN1^52,53^. In this study, melatonin promotes lipolysis via up-regulating ATGL, HSL, and PLIN1 expression. This observation is consistent with the finding reported by Kato *et al*. (2015) in 3T3-L1 cells^23^. Recent studies demonstrate that HSL and ATGL are direct target genes of PPARγ^52,53^. Thus, the up-regulation of HSL and ATGL could be directly mediated by the markedly increased expression of PPARγ in this study^54,55^. Interestingly, 1 mM melatonin significantly elevated the TAG level and led to larger lipid droplets, which is in consistent with the previous studies^56,57^. Weiszenstein *et al*.^56^ reported that mild hypoxia stimulated lipolysis and lipogenesis, and resulted in larger lipid droplets in 3T3-L1 cells^56^; Ju *et al*. (2011) found that estrogen-related receptor α significantly increased adipocytes differentiation, TAG accumulation and glycerol release in porcine adipocytes^57^. In adipocytes, lipid droplet size depends on the balance of lipogenesis and lipolysis, and triglycerides accumulate in adipocytes when lipogenesis exceeds lipolysis^57–59^. Lipolysis influences triglyceride accumulation via two mechanisms. First, lipolysis capacity is reduced, and triglyceride accumulation is subsequently promoted. Second, lipolysis supplies more free fatty acid (FFA) as a substrate for increased turnover of the cellular triglyceride pool during adipocyte differentiation^57,58^. These results indicate that melatonin can increase lipolysis to supply more FFA for triglyceride synthesis.

Current evidence indicates that ROS promotes adipocyte differentiation in mesenchymal stem cells and 3T3-L1 cells^60–62^. However, numerous *in vivo* and *in vitro* studies report contradictory effects of ROS on adipocyte differentiation and triglyceride accumulation^60,63,64^. Melatonin is a powerful antioxidant^65^ and increases antioxidant enzymes activities in 3T3-L1 preadipocytes^66,67^. In this study, melatonin significantly reduced intracellular ROS levels, up-regulated the expression levels and activities of antioxidant genes (SOD1 and Gpx4) and promoted high TAG accumulation in BIPs, which is consistent with previous studies in 3T3-L1and hMSCs^47,66,67^. The findings suggest that melatonin promotes adipocyte differentiation and TAG accumulation via decreasing intracellular ROS levels.

In mammals, numerous physiological roles of melatonin are mediated via activation of two high-affinity G protein-coupled receptors, MT1 and MT2^28,29^. MT1 and MT2 mediate the physiological role of melatonin in a manner dependent on melatonin concentration and exposure time, as well as cell type^39,40^. We found co-expression of MT1 and MT2 in BIPs and reported that MT2 expression exhibited significant differences during BIPs differentiation. Furthermore, 1 mM melatonin significantly increased the expression of MT2 at the mRNA and protein levels during BIPs differentiation. This result indicates that the effect of melatonin on adipogenesis, lipolysis, and redox status in BIPs may be mediated by MT2. Further studies determined that MT2, but not MT1, mediates the physiological role of melatonin in differentiation, lipolysis, and redox status in BIPs, given that luzindole and 4P-PDOT block these effects. These results are consistent with previous studies in which MT2 seemed to be more involved in the regulation of the redox status of 3T3-L1 cells^66,67^.

## Conclusion

In brief, we demonstrate for the first time that melatonin promotes the differentiation of BIPs into adipocytes with large lipid droplets by increasing the expression levels of PPARγ, C/EBPβ, and C/EBPα via a specific melatonin receptor, namely MT2. Formation of these large lipid droplets is likely due to the rate of lipogenesis exceeding the rate of lipolysis, as well as increased basal lipolysis supplying more FFA as a substrate for TAG synthesis via the MT2 receptor. In addition, melatonin reduces intracellular ROS levels by increasing the gene expression and activities of antioxidant enzymes via the MT2 receptor. These results suggest that melatonin promotes TAG accumulation via MT2 receptor during differentiation in BIPs and improves the overall understanding of the regulatory mechanisms of melatonin on adipogenesis.

## Materials and Methods

### Ethics statement

This study was conducted in strict accordance with the Regulations for the Administration of Affairs Concerning Experimental Animals (Ministry of Science and Technology, China, revised 2004). The protocol was approved by the Committee on the Ethics of Animal Experiments of the Laboratory Animals of Northwest A&F University. The cattle were raised at the National Beef Cattle Improvement Centre (Yangling, China). Animal slaughter was conducted humanely as necessary to alleviate suffering.

### Cell culture

Intramuscular adipose tissue was isolated from sternomandibularis muscle was collected from 12 Qinchuan steers (24-week-old) and every 4 steers were mixed. BIPs were isolated under sterile conditions using a modification of the method described by Wan *et al*.^68^ and Aso *et al*.^69^. Briefly, aseptically isolated intramuscular fat tissue from sternomandibularis muscle then washed with PBS and cut into approximately 1 mm^3^ sections. The tissue was digested with 0.1% type I collagenase (Sigma, St. Louis, MO, USA) for 1 h at 37 °C in a water bath. Following incubation, the isolation buffers containing the intramuscular fat tissue samples were passed through a 100- and 70-μm nylon mesh successively and then centrifuged for 5 min at 1,500 rpm. Supernatant was removed and the cell pellet was suspended in 20 mL of warm (37 °C) DMEM containing 10% fetal bovine serum (FBS). The pure BIPs were isolated after 2 to 3 passages and transferred to a six-well plate and adjusted to 1 × 10^6^ cells/well using growth medium containing DMEM/F12 and 10% fetal bovine serum (FBS, Millipore Sigma, St. Louis, MO) and maintained in the same medium for 2 to 3 days at 37 °C and 5% CO_2_. When cells grew to 80% confluency, differentiation was then induced with culture medium (10%FBS, DMEM/F12) containing 100 ng/ml dexamethasone, 0.5 mM IBMX, and 5 µg/ml insulin (Millipore Sigma, St. Louis, MO) for 48 h followed by 5 µg/ml insulin alone for 6 additional days. To determine the effects during the course of BIPs differentiation, melatonin (Millipore Sigma, St. Louis, MO) at 1 nM, 100 nM, 10 µM or 1 mM was maintained in the cell cultures during the 8-day period based on previous studies^13,21–23,43^. After identifying the effective concentration of melatonin during differentiation of BIPs, the optimal melatonin concentration (1 mM) was selected throughout experiments, and non-supplemented melatonin was considered as control group. To identify which receptor is involved in these actions, 10 µM luzindole or 4P-PDOT supplemented with 1 mM melatonin or not was maintained in the cell cultures during the 8-day period based on previous study^22^. The experiments were done in three biological replicates and two technical replicates for each one of these.

### Detection of cell proliferation

Cell proliferation was detected using Cell Counting Kit-8 following the manufacturer’s instructions (Applygen Technologies, Beijing, China). Cultured BIPs in growth medium were digested by 0.25% trypsin (Millipore Sigma, St. Louis, MO) and resuspended in growth medium containing different concentrations of melatonin (1 nM, 100 nM, 10 µM or 1 mM) and then seeded at 4 × 10^3^ cells per 200 μL in a 96-well plate and cultured in an incubator with 5% CO_2_ and saturated humidity at 37 °C. After 24, 48 or 72 h of treatment, CCK-8 reagent was added to the cells to detect cell proliferation rates. After incubation at 37 °C for 1 h in the chamber, the BIPs proliferation rate was determined by measuring the light absorbance at 450 nm on a microplate reader (BioTek, Winooski, VT, USA).

### Total RNA extraction and real-time PCR

Total RNA was isolated from BIP using the RNAprep pure Cell/Tissue Kit (Tiangen Biotech, Beijing, China) according to the manufacturer’s instructions. First-strand cDNA was synthesized using a reverse transcription kit (Fermentas, Waltham, USA). Specific primers were designed using the Primer 5.0 software and synthesized by Sangon Biotech Co., Ltd. (Shanghai, China) based on the published sequences of PPARγ, C/EBPβ, C/EBPα, MT1, MT2, HSL, ATGL, PLIN1, SOD1, GPX4, β-actin, and glyceraldehyde-3-phosphate dehydrogenase (GAPDH) from GenBank (Table 1). Quantitative real-time PCR (RT-PCR) was performed using SYBR Premix EX Taq II (Takara) and a 7500 Real-Time PCR system (Applied Biosystems, Foster City, CA). A total 10 μL reaction mixture contained of 5 ng sample cDNA, 5 μM specific forward and reverse primers, and 5 μL SYBR Premix EX Taq. Normalization was performed using the house keeping genes β-actin and GAPDH as control, which have been used often as an internal control gene in recent studies of adipogenesis in BIP cell^70,71^. The relative expression results were obtained using the 2^−ΔΔCt^ method, which were first normalized to the geometric mean of two endogenous control genes^72^. The experiments were done in three biological replicates and two technical replicates for each one of these.

**Table 1:** Sequences of primer pairs and amplification conditions for real time PCR.

Gene	Primer sequences (5′-3′)	Annealing temperature	Amplicon size	Accession number
C/EBPβ	TTCCTCTCCGACCTCTTCTC	61 °C	74 bp	NM_176788
	CCAGACTCACGTAGCCGTACT			
PPARγ	CGTGGACCTTTCTATGATGGATG	61 °C	90 bp	NM_181024
	GATACAGGCTCCACTTTGATTGC			
C/EBPα	ATCTGCGAACACGAGACG	61 °C	69 bp	NM_176784
	CCAGGAACTCGTCGTTGAA			
HSL	CGGGGAGCACTACAAACGAAAC	61 °C	264 bp	NM_001080220
	GTCAGAGGCATTTCAAAGGCGA			
ATGL	TGCTGATTGCTATGAGTGTGCC	61 °C	101 bp	NM_001046005
	CCTCTTTGGAGTTGAAGTGGGT			
PLIN1	CAGAGACCGAGGAGAGCAA	61 °C	122 bp	NM_001083699
	CCACATCACGACTGAGACG			
MT1	CACAGCCTCAGATACGACAAG	61 °C	245 bp	XM_614283
	GCCCAGATTCTCAGGTAACAG			
MT2	TGGTCCTTCTGCCCAACTT	61 °C	155 bp	NM_001206907
	CAGGTAGCAGAAACACACAAC			
GPX4	TGTGCTCGCTCCATGCACGA	61 °C	224 bp	NM_174770
	CCTGGCTCCTGCCTCCCAA			
SOD1	GCTGTACCAGTGCAGGTCCTCA	61 °C	228 bp	NM_000454
	CATTTCCACCTCTGCCCAAGTC			
β-actin	CACCAACTGGGACGACAT	61 °C	202 bp	NM_173979
	ATACAGGGACAGCACAGC			
GAPDH	CCAACGTGTCTGTTGTGGAT	61 °C	80 bp	NM_001034034
	CTGCTTCACCACCTTCTTGA			

### Western blotting

For western blotting, cells were collected and lysed in RIPA buffer (Solarbio, Beijing, China) supplemented with PMSF (Pierce, Rockford, IL). Total protein concentrations were determined by the BCA assay (Pierce, Rockford, USA). Proteins were separated on 12% polyacrylamide gels and transferred onto PVDF membranes (Millipore, Bedford, MA). The membrane was treated with a blocking buffer (5% dried nonfat milk in PBS containing 0.1% Tween 20) for 1 h at room temperature and incubated overnight at 4 °C with the following antibodies: PPARγ (Cat#: bs-0530R; Bioss Inc., Woburn, MA; 1:250), MT1 (Cat#: bs-0027R; Bioss Inc., Woburn, MA; 1:250), MT2 (Cat#:bs-23279R; Bioss Inc., Woburn, MA; 1:250), HSL (Cat#:SAB4501762; Sigma, USA; 1:200), and β-actin (Cat#:bs-0061R; Bioss Inc., Woburn, MA; 1:1000). Then, the membranes were washed three times with PBS-T and incubated for 1 h at room temperature with 3500-fold diluted HRP labeled goat or mouse anti-rabbit IgG (Santa Cruz Biotechnology, Inc., USA). Immunoreactive bands were detected using an enhanced chemiluminescence detection kit (Amersham Biosciences, Piscataway, NJ, USA) and scanned on a chemiluminescent imaging system (MFChemiBIS3.2, DNR Bio-Imaging Systems, Ltd., Jerusalem, Israel). The photos of the blots were captured and gel documentation of the density of each band was determined using Image J software (National Institutes of Health, Bethesda, MD, USA). The ratio of each band/β-actin was considered as the expression level of the target protein. The experiments were done in three biological replicates and three technical replicates for each one of these.

### Oil red O staining

The BIPs that were differentiated for 8 days were washed 3 times in PBS. Cells were fixed in 10% (vol/vol) paraformaldehyde for 1 h and then washed again with PBS. The lipid droplets in the cells were stained with 5% oil red O in isopropanol for 40 min, washed with PBS and examined by fluorescence microscope (Nikon, Tokyo, Japan). The average size of 100 lipid droplets per sample was measured on Image J software (version 1.47 v; NIH, Bethesda, MD, USA) using a modification of the method described by Deutsch *et al*.^73^. Each visible oil red O stained droplet was manually traced using the circle tool of Image J software, which recorded the diameter of each droplet.

### Triacylglycerol assay

The levels of cellular TAG in BIPs that were differentiated for 8 days were assayed using the tissue triglyceride assay kit (Applygen Technologies, Beijing, China). Total protein concentrations were determined by the BCA assay (Pierce, Rockford, USA). All of the experiments were performed according to the manufacturer’s recommended protocols. The values obtained were normalized to the total cellular protein content and were expressed as micrograms per milligram of protein.

### Lipolysis

BIPs that had differentiated for 8 days were incubated for 2 h in fresh medium with or without 10 mM isoproterenol. The medium was collected to detect the glycerol release, a measure of lipolysis, using the Adipolysis Assay Kit (Cayman, Ann Arbor, MI, USA) according to the manufacturer’s instructions. Lipolysis was calculated in nmol per mg total protein per h.

### Reactive oxygen species determination

The ROS level in the intramuscular adipocytes was measured usingthe 2,7-dichlorofluorescin diacetate (DCFH-DA) assay (Applygen Technologies, Beijing, China). BIPs that had differentiated for 8 days were incubated in growth medium containing 10 μM DEHF-DA at 37 °C for 45 min. After washing three times in PBS, fluorescent emissions from the intramuscular adipocytes were detected using a Nikon Eclipse Ti-S microscope equipped with a198 Nikon DS-Ri1 digital camera (Nikon, Tokyo, Japan). The recorded fluorescent images were analyzed using the Image J software (version 1.47 v; NIH, Bethesda, MD, USA). The background fluorescent values were subtracted from the final values before analyzing the statistical difference among the groups. The experiments were done in three biological replicates and three technical replicates for each one of these.

### Determination of antioxidant enzymes activities

The activities of antioxidant enzymes were measured using a SOD and GPX assay kit (Jiangcheng Bioengineering Nanjing, China). The intracellular GPX assay mainly included enzymatic reaction and color reaction. Intracellular SOD in cell cytosol was measured using the xanthine oxidation method. The spectrophotometric absorbance was assessed at 412 and 450 nm for GPX and SOD, respectively, on a microplate reader (BioTek, Winooski, VT, USA) according to the manufacturer’s instructions.

### Data analysis

All data were expressed as mean ± S.E.M. from at least three independent experiments. Group data for multiple comparisons were analyzed by ANOVA using a general linear model procedure followed by Tukey’s test using the SPSS statistics 17 software (SPSS Inc., Chicago, IL). *P* < 0.05 was considered to indicate a significant difference and *P* < 0.01 was considered to be a highly significant difference.
